# Dysfunction of the hypothalamic-pituitary adrenal axis and its influence on aging: the role of the hypothalamus

**DOI:** 10.1038/s41598-023-33922-5

**Published:** 2023-04-27

**Authors:** Melanie Spindler, Marco Palombo, Hui Zhang, Christiane M. Thiel

**Affiliations:** 1 Biological Psychology, Department of Psychology, School of Medicine and Health Sciences, Carl von Ossietzky Universität Oldenburg, 26129 Oldenburg, Germany; 2 Cluster of Excellence “Hearing4all”, Carl Von Ossietzky Universität Oldenburg, 26129 Oldenburg, Germany; 3 Cardiff University Brain Research Imaging Centre (CUBRIC), School of Psychology & School of Computer Science and Informatics, Cardiff University, Cardiff, UK; 4 Department of Computer Science and Centre for Medical Image Computing (CMIC), University College London (UCL), London, UK; 5 Research Centre Neurosensory Science, Carl von Ossietzky Universität Oldenburg, 26129 Oldenburg, Germany

**Keywords:** Neuroscience, Anatomy, Diseases, Endocrinology

## Abstract

As part of the hypothalamic-pituitary adrenal (HPA) axis, the hypothalamus exerts pivotal influence on metabolic and endocrine homeostasis. With age, these processes are subject to considerable change, resulting in increased prevalence of physical disability and cardiac disorders. Yet, research on the aging human hypothalamus is lacking. To assess detailed hypothalamic microstructure in middle adulthood, 39 healthy participants (35–65 years) underwent comprehensive structural magnetic resonance imaging. In addition, we studied HPA axis dysfunction proxied by hair cortisol and waist circumference as potential risk factors for hypothalamic alterations. We provide first evidence of regionally different hypothalamic microstructure, with age effects in its anterior–superior subunit, a critical area for HPA axis regulation. Further, we report that waist circumference was related to increased free water and decreased iron content in this region. In age, hair cortisol was additionally associated with free water content, such that older participants with higher cortisol levels were more vulnerable to free water content increase than younger participants. Overall, our results suggest no general age-related decline in hypothalamic microstructure. Instead, older individuals could be more susceptible to risk factors of hypothalamic decline especially in the anterior–superior subregion, including HPA axis dysfunction, indicating the importance of endocrine and stress management in age.

## Introduction

Beginning in middle adulthood, the human body undergoes considerable changes, including downregulation of the endocrine system and hormone production, physiological decline, and alterations of energy homeostasis, possibly leading to muscle loss and obesity^1,2^. To maintain metabolic functioning throughout the lifespan, the hypothalamus, a small diencephalic region, is essential. It controls body homeostasis for instance through hormone secretion, thereby regulating the autonomic nervous system, circadian rhythm, and stress response^3,4^. It is well established that hypothalamic damage is associated with dysfunction of the hypothalamic–pituitary–adrenal axis (HPA axis), for example through overnutrition and chronic stress, resulting in metabolic (e.g., obesity and type II diabetes) and mood disorders^5,6^. By contributing to microinflammation, these are proposed to drive cellular aging processes associated with functional decline in age and age-related disease^7^. In animals, proinflammatory mediators like nuclear factor-κB were shown to promote oxidative stress leading to cell death and are associated with iron accumulation in different areas of the brain^8–11^. Meanwhile, iron deposition can itself contribute to inflammation in the brain^12^. Overall, inflammation, cell death, and iron accumulation are associated with neurodegenerative diseases, such as Alzheimer’s and Parkinson’s disease, but also accompany the healthy aging process^13–15^. Traditionally, these markers are examined invasively in animal studies. However, in recent years, non-invasive neuroimaging techniques have been developed to assess inflammation- and iron-related tissue microstructure in-vivo in humans. Therefore, the investigation of proinflammatory mediators and iron accumulation is in the focus of current research to counteract age-related decline.

Even though the hypothalamus is involved in many functions undergoing change throughout life, its role in aging is yet to be well understood. Studies in rodents have demonstrated that the lifespan could be increased or decreased by manipulation of an inflammatory immune pathway in the mediobasal hypothalamus, thus supporting the role of hypothalamic microinflammation in age^16,17^. In humans, to date, knowledge on changes of the hypothalamus in age is restricted to ex-vivo investigations. Here, it was shown that nuclei of the hypothalamus are differentially affected, some showing decreases in cell count with age, whereas others maintain their structure throughout the lifespan, or even become increasingly active in older age^18^. However, age-related iron accumulation or inflammation of individual hypothalamic regions remains to be investigated. To elucidate neural correlates of metabolic disorders and stress, the paraventricular nucleus (PVN) is of particular interest, since it plays a central role in many homeostatic regulations^6^. In aging, microstructural changes throughout the brain have often been observed to follow an inverted u-shaped distribution, showing peak microstructure in early adulthood, followed by a decline starting at about 35–40 years^19,20^, but evidence on the development of the hypothalamus with age is lacking.

In the past years, magnetic resonance imaging (MRI) has emerged as the state-of-the-art method for non-invasive analysis of the human hypothalamus in vivo, and modern MRI techniques have enabled the quantification of tissue properties to assess its microstructure. These include microstructural parameters derived from quantitative MRI (qMRI), such as the multi-compartment model neurite orientation dispersion and density imaging (NODDI) derived from diffusion imaging, and multiparameter mapping (MPM), providing for example measures of neurite density, dendritic dispersion, free water content (NODDI), and iron content (MPM). While such approaches have been successfully used to detect microstructural alterations related to aging across different regions of the brain, microstructural imaging of the hypothalamus is challenging. First, the hypothalamic region is heterogenous, containing crossing white matter tracts (WM) and cerebrospinal fluid (CSF) from the third ventricle, which presents a confounding influence that needs to be considered^21^. Second, the approximately 15 hypothalamic nuclei are involved in different metabolic, affective, and cognitive functions, encouraging a division into anatomically and functionally reasonable components, to help understand differential effects of aging on microstructure of hypothalamic subunits. This notion is supported by previous work in our group, where we demonstrated that of four different subunits, only in the anterior–superior subunit, hypothalamic mean diffusivity (MD), an unspecific marker of tissue microstructure, was significantly related to obesity, operationalized by the body-mass index (BMI).

Owing to the complex, heterogenous structure of the hypothalamus, the first goal of this study is to anatomically characterize the individual hypothalamic subregions in a healthy middle-aged to elderly sample (35–65 years) to assess subregion-specific hypothalamic microstructure in detail and in relation to age. We quantify microstructure using MPM and NODDI to explore the influence of age on markers of iron and water content, neurite density, and dendritic dispersion.

Secondly, we aim to build upon our previous research linking BMI to anterior–superior hypothalamic MD in a sample of young healthy adults, hinting towards obesity-related inflammation. The anterior–superior hypothalamus is a critical region for body homeostasis and HPA axis regulation. It is comprised of the preoptic nucleus and PVN, which is responsible for autonomic control, including the stress response through regulation of the HPA axis. In the PVN, corticotropin-releasing hormone is released in response to stress, which controls cortisol secretion, the main stress hormone. Through inhibitory feedback mechanisms involving the hippocampus, amygdala, and prefrontal cortex, cortisol production is then downregulated to achieve homeostasis. It is proposed that chronic stress and obesity are associated with a desensitized HPA axis, which is reflected by faulty inhibitory feedback, atrophy and loss of glucocorticoid receptors in the above-mentioned brain regions^22,23^. Hence, we hypothesize that HPA axis dysfunction would affect anterior–superior hypothalamic microstructure by facilitation of inflammation (i.e., increased free water fraction (FWF) measured with NODDI) and iron accumulation (i.e., the effective transverse relaxation rate (R2*) measured with MPM) with age. Thus, we investigate HPA axis dysfunction (i.e., obesity and long-term stress) as a potential driving factor for inflammatory processes and iron accumulation in the anterior–superior hypothalamus during middle adulthood. Here, we assume that older subjects are more vulnerable to microstructural alterations as a result of HPA axis dysfunction as compared to younger individuals. Overall, the results of this study can help to uncover the role of detailed hypothalamic microstructure and its related functions with age.

## Results

Table 1 depicts a summary of the participant characteristics. Measures of interest include age, sex, waist circumference, and cortisol. The remaining parameters are displayed for descriptive purposes only.

**Table 1:** Participant characteristics.

Participant characteristics	n/Range	M ± SD
Age	35–65	50.2 ± 8.4
Sex (Male/Female)	17/22	–
Education (years)	9–19	14.8 ± 3.4
Waist circumference (cm)	74–124	94.9 ± 12.5
BMI (kg/m^2^)	18.8–40.0	25.0 ± 4.5
Thyroid medication (yes/no)	5/34	–
Blood pressure medication (yes/no)	6/33	–
Systolic blood pressure	90–167	122 ± 17
Diastolic blood pressure	57–99	75 ± 9
High blood pressure (yes/no)	8/31	–
Cortisol (pg/mg; n = 38)	1.4–15.6	5.5 ± 3.2

Education was measured in years until the highest achieved degree. High blood pressure was defined as systolic pressure ≥ 140, and/or diastolic pressure ≥ 90.

### Hypothalamic subunit changes with age

In a first step, we investigated whether age or sex impact on neurite density, orientation dispersion, free water fraction, or iron content in each of the four hypothalamic subunits, as well as possible age-related differences in subunit-specific microstructure (n = 39). For this purpose, four linear mixed effects models were used, predicting microstructure with the variables age, sex, subunit, and the age × subunit interaction. To determine whether our predictors significantly improve the models, we computed likelihood ratio tests assessing model significance between the intercept and full models (Table 2).

**Table 2:** Likelihood ratio test results comparing the linear mixed effect models comprised of the intercept of the fixed and random effects (Intercept), and the full model (Full) testing microstructural differences in hypothalamic subunits with age (neurite density index (NDI), orientation dispersion index (ODI), free water fraction (FWF), effective transverse relaxation rate (R2*); Akaike information criterion (AIC), Bayesian information criterion (BIC)).

	NDI		ODI		FWF		R2*	
	Intercept	Full	Intercept	Full	Intercept	Full	Intercept	Full
Log likelihood	− 219.34	− 179.63	− 220.46	− 189.84	− 220.85	− 122.61	− 211.94	− 194.47
χ^2^	–	79.416***	–	61.224***	–	196.49***	–	34.95***
AIC	444.68	381.27	446.91	401.69	447.71	267.22	429.88	410.93
BIC	453.83	414.82	456.06	435.23	456.86	300.77	439.03	444.48

*** *p* < .001.

Significant values are in [bold].

**Figure 1:**
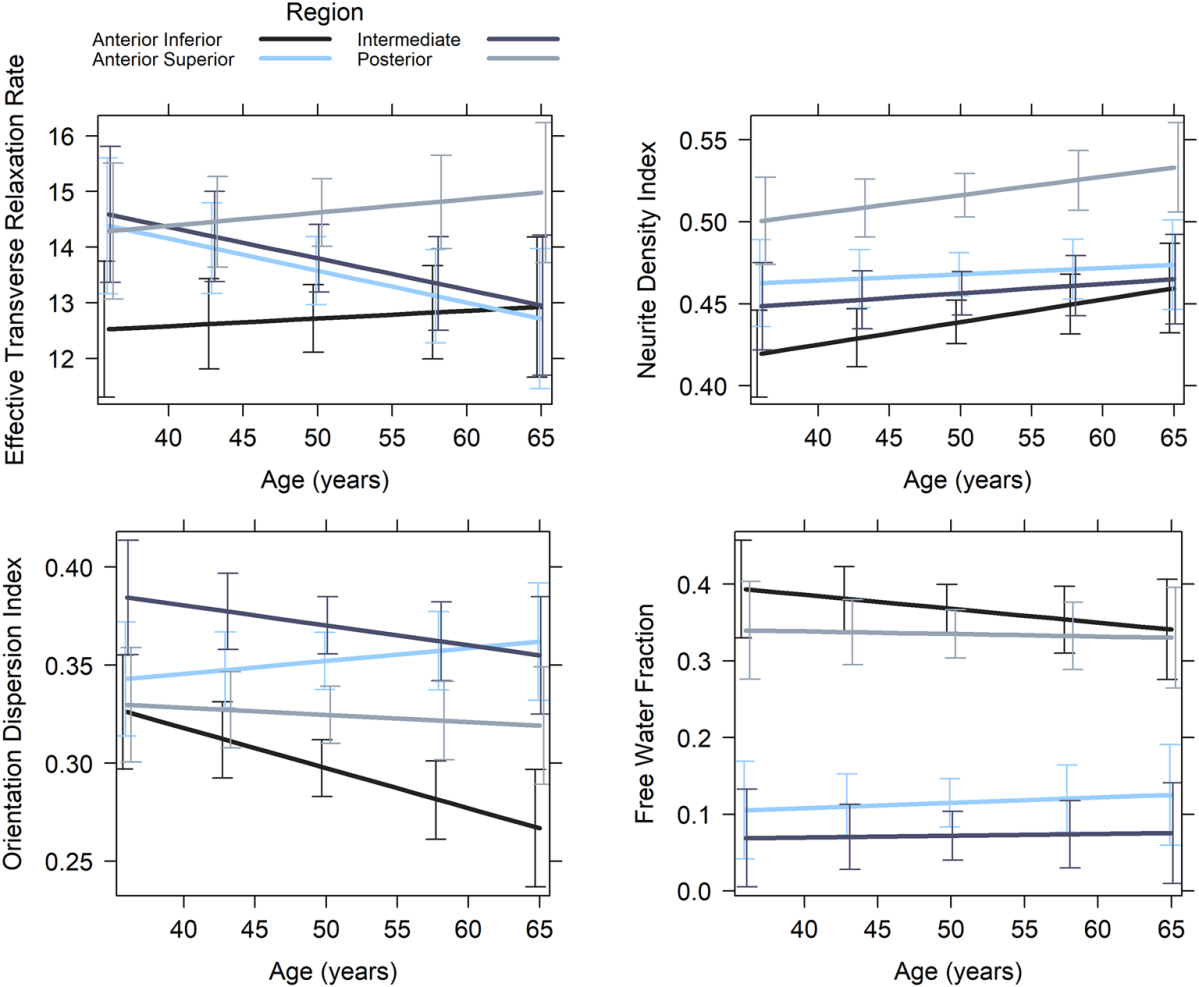
Hypothalamic microstructure across subunits in association with age investigated with linear mixed effects models. Error bars depict the 95% confidence interval.

Afterwards, we tested the full linear mixed effects models to assess differences in microstructure across subunits and how this microstructural composition varies across age, while controlling for the fixed effect of sex and the subject-specific random intercept. Results suggest that hypothalamic subunits differ in their microstructural composition across all metrics. In addition, we observed an increase of the orientation dispersion index with age in the anterior–superior subunit, whereas microstructure in the other subunits was not significantly associated with age (Table 3, Fig. 1). Hence, our results suggest that all hypothalamic subunits are distinguishable by measures reflecting neurite density, dendritic dispersion, free water content, and iron content. However, only dendritic dispersion predominantly in the anterior–superior subunit undergoes age-associated changes.

**Table 3:** Linear mixed effects on hypothalamic microstructure (neurite density index (NDI), orientation dispersion index (ODI), free water fraction (FWF), and effective transverse relaxation rate (R2*)) between subunits (anterior-inferior (A-Inf), anterior–superior (A-sup), intermediate (Int), posterior (Post)) and across age while controlling for sex.

	NDI		ODI		FWF		R2*	
	β (SE)	t	β (SE)	t	β (SE)	t	β (SE)	t
Intercept	− 0.42 (0.16)	− 2.582*	− 0.58 (0.17)	− 3.464***	0.84 (0.13)	6.408***	− 0.34 (0.20)	− 1.736
Sex (Female)	− 0.30 (0.18)	− 1.662	− 0.23 (0.18)	− 1.326	0.06 (0.15)	0.398	− 0.21 (0.23)	− 0.906
Age	0.22 (0.13)	1.681	− 0.31 (0.14)	− 2.281*	− 0.09 (0.10)	− 0.922	0.06 (0.15)	0.366
Subunit (-A-Inf)								
A-Sup	0.55 (0.15)	3.561***	1.01 (0.17)	5.907***	− 1.53 (0.10)	− 15.414***	0.41 (0.16)	2.498*
Int	0.33 (0.15)	2.142*	1.33 (0.17)	7.805***	− 1.79 (0.10)	− 18.037***	0.51 (0.16)	3.154**
Post	1.47 (0.15)	9.520***	0.50 (0.17)	2.947**	− 0.20 (0.10)	− 1.989 *	0.92 (0.16)	5.647***
Age × Subunit (-A-Inf)								
Age: A-Sup	− 0.16 (0.16)	− 1.016	0.41 (0.17)	2.404*	0.13 (0.10)	1.272	− 0.29 (0.16)	− 1.761
Age: Int	− 0.13 (0.16)	− 0.827	0.16 (0.17)	0.916	0.10 (0.10)	1.036	− 0.28 (0.16)	− 1.730
Age: Post	− 0.04 (0.16)	− 0.259	0.26 (0.17)	1.498	0.08 (0.10)	0.753	0.04 (0.16)	0.249

*** *p* < .001, ** *p* < .01, * *p* < .05.

Significant values are in [bold].

### Influence of long-term stress on anterior–superior microstructure

In this study, hair cortisol and waist circumference were treated as proxies for HPA axis dysfunction, suggesting long-term stress and obesity. The microstructural parameters FWF and R2* are related to tissue water and iron content and were used to depict probable microstructural alterations associated with this HPA axis dysfunction. Given the role of the anterior–superior subunit in maintaining HPA axis regulation, we investigated whether FWF and R2* in this subunit are related to waist circumference, hair cortisol, and age. To that aim, two robust linear regression models were tested (n = 38).

For FWF, we found a significant effect of waist circumference, suggesting increased water content associated with higher waist circumference. In addition, we observed a significant interaction of age × cortisol, suggesting that individuals with higher long-term cortisol showed increased free water content in age, as opposed to individuals with low cortisol markers (R^2^ = 0.361, adj. R^2^ = 0.261, χ^2^(5) = 17.809, *p* = 0.003). For R2*, a significant effect of waist circumference was observed with lower iron concentrations related to higher waist circumference, but no effect of age, cortisol, or their interaction (R^2^ = 0.268, adj. R^2^ = 0.153, χ^2^(5) = 13.89, *p* = 0.016).

**Figure 2:**
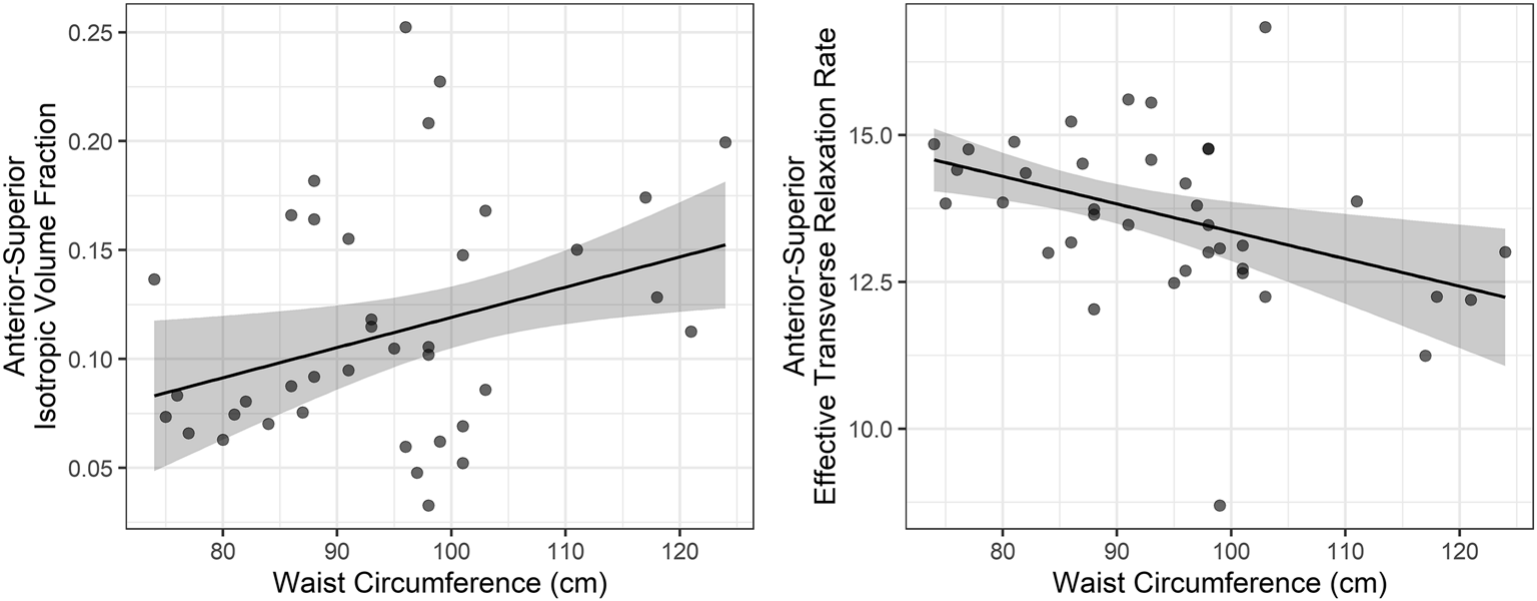
Scatterplots showing the associations between waist circumference and anterior–superior hypothalamic free water fraction (left), and effective transverse relaxation rate, indexing iron content (right).

**Figure 3:**
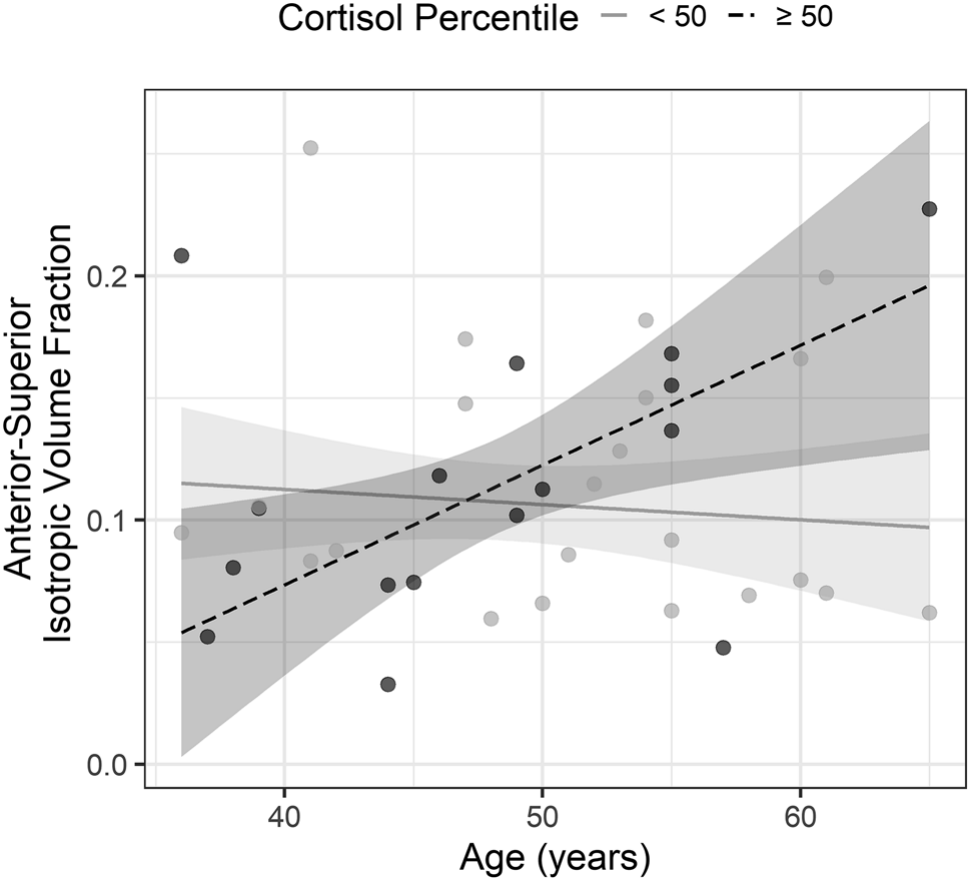
Scatterplot showing the association between anterior–superior hypothalamic free water fraction, age, and long-term cortisol. To display the interaction effect between age and cortisol, we divided the participants into two groups based on their age- and sex-dependent cortisol percentile (< 50 and ≥ 50 percentile).

Hence these results suggest that waist circumference is associated both with increased free water content and decreased iron content (Fig. 2), but that only free water content was influenced by an age-dependent effect of long-term cortisol (Table 4). Here, participants with a higher age- and sex-specific cortisol percentile (≥ 50, n = 16) showed an increase in anterior–superior free water content, as compared to participants with lower age- and sex-specific cortisol percentile (< 50, n = 22), indicating a stronger influence of long-term stress in older as compared to younger subjects (Fig. 3). The interaction of waist circumference × cortisol was not significant in both models, suggesting no moderating effect.

**Table 4:** Results of two robust multiple linear regression analyses predicting free water fraction (top) and effective transverse relaxation rate (iron content, bottom) in the anterior–superior hypothalamus.

Predictor	Standardized β	Standard error	t-value	*p* value
**Free water fraction**				
Intercept	− .071	.147	− 0.482	.633
Age	.190	.152	1.252	.220
Cortisol (log trans.)	.051	.205	0.250	.804
Waist circumference	.319	.129	2.477	**.019**
Age × Cortisol	− .420	.166	− 2.534	**.016**
Waist circumference × Cortisol	.133	.145	0.911	.369
**Effective transverse relaxation rate**				
Intercept	.016	.135	0.116	.909
Age	− .118	.182	− 0.647	.522
Cortisol (log trans.)	− .143	.392	− 0.366	.717
Waist circumference	− .409	.132	− 3.090	**.004**
Age × Cortisol	− .070	.409	− 0.172	.864
Waist circumference × Cortisol	.018	.091	0.195	.847

Significant values are in [bold].

## Discussion

This study aimed to investigate effects of age and long-term stress on detailed subunit-specific hypothalamic microstructure in vivo in middle-aged to elderly individuals. To quantify microstructure, NODDI and MPM were employed to obtain estimates of neurite density, dendritic complexity, free water, and iron content.

We found that microstructural composition differed across hypothalamic subunits. For example, we observed that the orientation dispersion index (ODI), a measure of dendritic branching, is highest in the intermediate subunit and lowest in the anterior-inferior subunit. This is in accordance with previous research, as large parts of the anterior-inferior area are known to project to the pituitary, whereas the connections of the lateral (i.e., intermediate) region are more strongly dispersed^24^. These results suggest that hypothalamic parcellation is beneficial for precise quantitative assessment of hypothalamic microstructure. In addition, it encourages the investigation of microstructural differences in health and disease to identify possible MRI-based biomarkers. However, we found mostly stable microstructural properties from the fourth up to the seventh decade of life. Only the orientation dispersion index in the anterior–superior subunit was positively associated with age, possibly reflecting more inefficient dendritic connections and processing in this area with increasing age. This finding emphasizes the relevance of the anterior–superior hypothalamus in age-related decline. However, surprisingly, other subunits and microstructural metrics showed no significant associations with age.

One possible explanation for this finding is that brain regions that are developed early in life, such as the hypothalamus, are more resilient towards age-related decline than brain regions with high rates of neural plasticity throughout adulthood^25^. Additionally, even though we applied a rigorous procedure correcting for partial volume contaminations from confounding WM and CSF, we observed hypothalamic microstructure to be highly variable. Hence, slow deterioration in age is likely to be overlooked in our sample, especially since our small sample size does not allow us to reliably identify small effects. Future studies are needed to examine this in more detail.

Next, we investigated whether HPA axis functioning drives the observed variability in hypothalamic microstructure, and whether this influence is dependent on age. Here, we hypothesized that older individuals would be less able to cope with detrimental influences of persistent stress on the immune system and therefore show stronger decline in microstructure, which would translate into inflammation and iron accumulation^26^. We operationalized persistent stress by hair cortisol concentrations, a reliable and sensitive method to assess HPA axis dysfunction^27^, and complemented our analyses by obesity (i.e., waist circumference). As the anterior–superior subunit of the hypothalamus has a pivotal role in HPA axis regulation, and since we previously found that obesity (i.e., BMI) was only related to mean diffusivity in this subunit in a large sample^28^, we restricted our analysis to this region.

Our results provide first evidence that participants with higher long-term stress display increased free water in the anterior–superior hypothalamus, potentially indicative of inflammation^29–31^, and that this increase is dependent on age. Here, older, stressed participants had higher free water fraction as compared to non-stressed participants of similar age. In addition, we were able to strengthen previous findings^28^ by showing an increase in water content in individuals with higher waist circumference, indexing obesity. Both results suggest that hypothalamic deterioration could be a result of age-related vulnerability and HPA axis dysfunction.

In contrast, we found no effect of persistent stress on anterior–superior iron content, but surprisingly, we observed waist circumference to be negatively associated with iron levels. Both iron accumulation and iron deficiency are known to have adverse effects on the brain. As previous research has pointed out, iron exchange out of the brain is slow. It is therefore unlikely that lower hypothalamic iron levels are explainable by adult dietary habits^32^. Nevertheless, events in early life, such as infant nutritional deficiency could result in long-term brain iron deficiency, leading to dysfunction of mitochondria, that in turn promote oxidative stress and inflammation^33^. However, this could not be examined here. Finally, no interaction effects were observed between cortisol levels and waist circumference related to iron or free water content. This suggests that in our sample, the effect of waist circumference on microstructure is not moderated by cortisol levels, or vice versa. Taken together, our results point towards an important role of HPA axis function on hypothalamic microstructure.

In the past, greater amount of free water has been attributed to tissue damage and edema, possibly reflecting microinflammation^34,35^. However, other underlying processes, such as cell death or demyelination in the absence of inflammation could lead to the same observed changes. The observed increase in overall diffusion (mean diffusivity^28^), or extracellular content (free water fraction) can therefore not be directly linked to inflammation-induced tissue degeneration. However, rodent studies have pointed towards the important role of inflammatory regulatory pathways in the hypothalamus and their implication for metabolic disorders and aging^8,36,37^, thus supporting our findings. Nevertheless, future studies with larger samples able to integrate multiple metrics are needed to gain a deeper understanding of in-vivo hypothalamic microstructure in age and associated disorders. The associations between HPA axis regulation, obesity, stress, and aging are highly complex, and our study might be underpowered to detect relevant associations. Thus, our results need to be interpreted with caution and future research is needed encompassing more, relevant features related to hypothalamic microstructure.

For example, HPA axis dysfunction is also considered one of the main mechanisms underlying the development of mood disorders, including anxiety, major depressive, and bipolar disorder, but so far, research has obtained mixed results regarding the influence of hypothalamic structure. For example, one study found larger hypothalamic volume in individuals suffering from bipolar and depressive disorder as compared to healthy individuals^38^. In contrast, another study with a larger sample found no difference between hypothalamic volume in patients with major depressive disorder and healthy controls^39^. Therefore, in the future, more sensitive measures including NODDI imaging and MPM in addition to detailed segmentation could help better understanding the (micro)structural alterations in these disorders and their association with symptomatology.

## Conclusion

To conclude, we observed no general age-related decline in hypothalamic microstructure. Instead, we found an age-associated increase in dendritic arborization focused on the anterior–superior subunit. Here, microstructural properties were also related to obesity and long-term stress as markers for HPA axis dysfunction, and the effect of long-term stress was dependent on the age of the participants. Therefore, we argue that instead of a universal age-related decline, risk factors such as long-term stress can trigger adverse effects on anterior–superior hypothalamic microstructure in age possibly due to altered coping or compensatory mechanisms on a cellular level.

## Methods

### Participants

N = 40 healthy adult participants (n = 18 male, n = 22 female) aged between 35 and 65 took part in the current study. All participants were native German speakers recruited through local announcements between April and December 2021. The final sample size was affected by restrictions in data acquisition related to the COVID-19 pandemic but is comparable to sample sizes of previous research on brain microstructure in stress and aging^40–43^. Prior to participation, all subjects gave written informed consent to undergo the research assessment, and ethical approval of the study was granted by the University of Oldenburg ethics committee (Drs.EK/2020/062-01). Data acquisition was in accordance with the declaration of Helsinki^45^.

Exclusion criteria included diabetes type I or II, a history of or current neurological or psychiatric disease, current training for aerobic sports competitions such as marathons^46^, steroid medication during the last three months, and alcohol or substance abuse.

Two participants displayed considerably higher hair cortisol levels than average (> 3 SD from the mean) and were hence contacted again. One retrospectively reported drug abuse during the time of study. Therefore, this dataset was excluded for all analyses. The other participant reported no drug abuse, medication, or psychological problems that could have influenced the results. Therefore, the subject was excluded for cortisol analyses only. The final sample size was n = 38 for analyses involving hair cortisol, and n = 39 for the remaining analyses.

### Data acquisition

#### MRI

All participants underwent scanning on a Siemens 3 T Prisma scanner with a 64-channel head-neck coil for signal reception and a body coil for transmission. Total scanning time was approximately 60 min with eyes closed. T1-weighted images were obtained using a magnetization-prepared gradient-echo sequence (MP-RAGE). Based on the scanning protocols of Callaghan et al.^47^ and Tabelow et al.^48^, multi-echo 3D FLASH (fast low-angle shot) sequences were used to acquire qMRI (T1w, PDw, MTw). In addition, a spin-echo/stimulated echo imaging (SE/STE) sequence based on a SIEMENS product sequence was obtained, and two sensitivity maps from the head and body coil were acquired prior to each T1w, PDw, and MTw measurements. Diffusion data was acquired with the Center for Magnetic Resonance Research (CMRR), University of Minnesota, multiband diffusion sequence^49–51^. A three-shell diffusion scheme sampled on a whole sphere was employed with interleaved b = 300, 700, and 2000s/mm^2^ and 104 diffusion directions in total (8 for b = 300, 32 for b = 700, 64 for b = 2000). In addition, 13 b = 0 images were obtained interspersed throughout the sequence. Partial Fourier reconstruction was 6/8. Diffusion gradient duration and time were δ = 11.95 ms, and Δ = 38 ms, respectively. This data was acquired twice, directly after another, without slice gap. The multi-band factor was 3. All data was acquired from anterior to posterior phase-encoding (PE) direction, except for the single b0 image, which was acquired in posterior to anterior PE direction. A detailed report of imaging parameters is depicted in Table 5.

**Table 5:** Magnetic Resonance Imaging sequence parameters in the order of acquisition.

Sequence	Flip angle	FOV (mm)	Echo times (ms)	TR (ms)	No. slices	Voxel size (mm)
SE/STE	[90, 120, 60, 135, 45]	256 × 256	[14]	2000	18	4.0 × 4.0 × 5.0
T1w	21	256 × 256	[2.57, 5.82, 9.82, 13.9, 17.94, 21.98]	26	279	0.8 × 0.8 × 0.8
PDw	4	256 × 256	[2.57, 5.82, 9.82, 13.9, 17.94, 21.98]	26	279	0.8 × 0.8 × 0.8
MTw	6	256 × 256	[2.57, 5.82, 9.82, 13.9, 17.94, 21.98]	44	279	0.8 × 0.8 × 0.8
Sensitivity Head	6	256 × 256	1.99	4.1	44	4.0 × 4.0 × 5.0
Sensitivity Body	6	256 × 256	1.99	4.1	44	4.0 × 4.0 × 5.0
MP-RAGE	9	240 × 240	2.07	2000	224	0.75 × 0.75 × 0.75
b0	–	192 × 192	79	3427	75	1.5 × 1.5 × 1.5
Diffusion	–	192 × 192	79	3427	75	1.5 × 1.5 × 1.5

*SE/STE* spin-echo/stimulated echo imaging, *T1w* T1-weighted, *PDw* proton density-weighted, *MTw* magnetization transfer-weighted, *MP-RAGE* magnetization-prepared gradient-echo.

#### Physiological measures

Long-term cortisol derived from hair samples is a biomarker of HPA axis function and has been previously associated with lower reactivity to acute stress^52^ and higher ratings of chronic stress^53^. To obtain an estimate of cortisol secretion (pg/mg), the 3 cm hair segment closest to the scalp was analyzed, representing hair growth from the past three months (one month corresponding to approximately 1 cm hair growth). An at least 3 mm thick strand of hair was taken from as close to the scalp as possible, fixated with a string, and wrapped in aluminum foil. The samples were sent to the Department of Psychology laboratory of TU Dresden, Germany for analysis with liquid chromatography coupled with tandem mass spectrometry (LC–MS/MS)^54^. Prior to statistical analysis, hair cortisol levels were log transformed in accordance with previous research^55,56^.

We used waist circumference as additional proxy for HPA axis dysfunction, assessed with a tape measure on unclothed skin (cm). Compared with the BMI, waist circumference showed more consistent associations with serum inflammation markers in the literature^57,58^. Hence, even though both measures were acquired, only waist circumference was entered into the statistical analyses, given the high correlation of both measures (r = 0.820, *p* < 0.001).

Blood pressure was measured on a blood pressure monitor (SANITAS SBM 07) applied to the left, unclothed wrist for fast blood pressure monitoring (beats per minute (bpm)).

### MRI analysis

#### Diffusion MRI

Measures of neurite density, dendritic complexity, and free water content were derived from diffusion data. First, the raw data was denoised with MRTrix 3.0 *dwidenoise*, which has been shown to improve the quality of the diffusion data^59,60^. Since we observed no Gibbs ringing artifacts in the hypothalamic area, Gibbs ringing correction was not employed to not induce possible smoothing effects in the small region of interest. Diffusion data of the two runs was concatenated and corrected for susceptibility distortions with *topup* and *eddy* in the FMRIB Software Library (FSL) v. 6.0.4. For *topup* estimation, the raw data (before denoising) was used. Prior to eddy correction, the data was skull stripped using the FSL brain extraction tool. Using all shells, a NODDI model was fit using the NODDI toolbox running on Matlab 2020b^61^. NODDI provides measures of neurite density (NDI), dendritic complexity (ODI), and free water content (FWF).

#### Multiparameter maps

Iron content was derived from the multi-echo 3D FLASH sequences and MPMs were processed using the hMRI toolbox based on SPM12, running on Matlab 2020b. First, images for MPM calculation were reoriented to AC-PC orientation in midsagittal plane (*Autoreorient*). Afterwards, complete MPMs were calculated as part of the default hMRI pipeline (*createhMRI*), including magnetization transfer saturation (MT sat.), longitudinal relaxation rate (R1), proton density (PD), and R2*. To correct for radiofrequency sensitivity bias, head and body sensitivity maps were used individually for each contrast. Transmit field correction was performed based on the SE/STE sequence. For statistical analyses, we focus on R2* as a marker for iron content. The remaining MPMs did not enter into statistical analyses.

#### Segmentation

To obtain hypothalamic subunits, the MP-RAGE image was segmented into GM, WM, and CSF with SPM12 according to the ICBM152 2009b reference atlas. Subunits for the anterior–superior, anterior-inferior, intermediate (lateral), and posterior hypothalamus were obtained from the probabilistic atlas by Spindler, Özyurt and Thiel^28^ with a threshold of 20 to ensure reliable but complete representations of the hypothalamus. Afterwards, the subunits were automatically corrected for WM and CSF confounds that cannot be accounted for using the probabilistic atlas. CSF correction was applied on a voxel level with a threshold of CSF > 10% using the CSF tissue probability map. Confounding white matter from the optic tract, fornix, and mammillothalamic tract was automatically excluded using a previously published data-driven spectral clustering approach based on MT sat., R1, and R2* (k = 5, Spindler and Thiel^21^). Voxels identified as white matter were then excluded from the subunits. The final masks were visually inspected. In n = 4 cases with very small hypothalamus masks, white matter extraction resulted in voxels containing the mammillary bodies to be misclassified as white matter, which were manually corrected.

#### Registration

**Figure 4:**
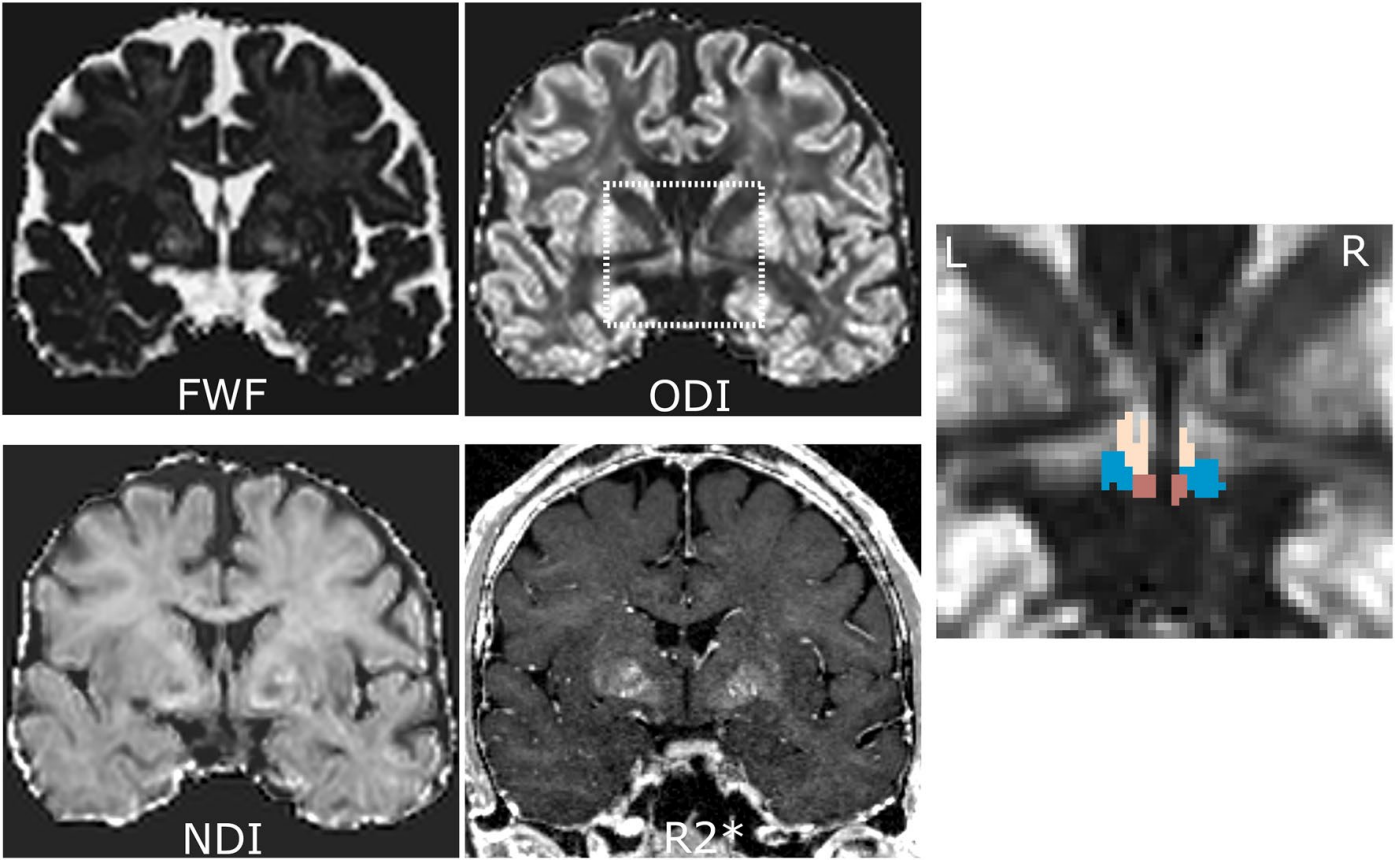
Exemplary dataset in native space displaying the four used MRI parameters in coronal view (FWF: free water fraction, ODI: orientation dispersion index corrected for FWF, NDI: neurite density index corrected for FWF, R2*: effective transverse relaxation rate), as well as the ODI image zoomed in on the hypothalamus area and overlaid with the hypothalamus masks after correction for confounding white matter (beige: superior, blue: intermediate, brown: posterior subunits). The inferior subunit is not visible on this slice.

The inverse deformations created in the segmentation were used to register the hypothalamic subunits to native space with nearest neighbor interpolation. In addition, NODDI parameter maps (NDI, ODI, FWF) were linearly registered and resliced to the MPMs with 4^th^ degree b-spline interpolation. For each hypothalamic subunit, mean R2*, NDI, ODI, and FWF were finally calculated, where NDI and ODI were computed using tissue-weighted means to further control partial volume contaminations from averaging within the masks^62^ (Fig. 4).

### Statistical analyses

All statistical analyses were performed with R v 4.1.1, using primarily the following packages: lme4 v.1.1.29^63^, lmerTest v.3.1.3^64^, and robustbase v.0.95.0^65^.

#### Hypothalamic subunit changes with age

To identify whether hypothalamic microstructure differs across subunits and to investigate possible differential effects with age, four maximum likelihood linear mixed effects models were tested (lme4). Linear mixed effects models allow the inclusion of random effects in the case of non-independence. Here, for each of the microstructural measures of interest, NDI, ODI, FWF, and R2*, we analyzed the effects of age, subunit (anterior-inferior, anterior–superior, intermediate, posterior), sex (female, male), and the age × subunit interaction as fixed effects. The participant intercept (1|subject) was entered as random effect to account for baseline differences in microstructure that affect all subunits, thus being (spatially) dependent. All metric variables were standardized before fitting the model (M = 0, SD = 1).

To test whether the predictors significantly improved the baseline model, likelihood ratio tests were performed between the intercept-only (Eq. 1) and the full model (Eq. 2). Here, β_0_ equals the intercept, β_1-4_ represent the coefficients for each of the effects, (1|subject) is the subject-specific random intercept, and ε the measurement error. The significance threshold of the likelihood ratio test (corrected for the four microstructural parameters) was *p* = 0.05/4 = 0.0125, following Bonferroni correction.

1 $${\text{Microstructure }} = {\upbeta }_{0} + \left( {1{\text{|subject}}} \right) + {\upvarepsilon }$$

2 $$\begin{gathered} {\text{Microstructure}} = {\upbeta }_{0} + {\upbeta }_{1} \left( {{\text{Sex}}} \right) + {\upbeta }_{2} \left( {{\text{Age}}} \right) + {\upbeta }_{3} \left( {{\text{Subunit}}} \right) \hfill \\ \quad \quad \quad \quad \quad \quad \quad + {\upbeta }_{4} \left( {{\text{Age }} \times {\text{Subunit}}} \right) + { }(1|{\text{subject}}) + {\upvarepsilon } \hfill \\ \end{gathered}$$

#### The anterior–superior hypothalamus in long-term stress

To investigate a possible association between anterior–superior hypothalamic water content, iron accumulation with age and long-term stress, two robust multiple linear models were tested with MM-estimator chain (lmrob) and compared against the intercept-only model. In small samples, outliers and high leverage points can significantly influence the results. Thus, we decided to use robust regression models instead of conventional multiple linear regression. Here, anterior–superior FWF and R2* were treated as dependent variables, which were predicted by age, waist circumference, hair cortisol level (log transform), and the interactions age × cortisol level and waist circumference × cortisol level (Eq. 3). All variables were standardized (M = 0, SD = 1). For interpretation of possible interactions, the participants were later divided into a low- and high-cortisol group (< 50 and ≥ 50 percentile, respectively), based on sex- and age-specific norm values obtained through personal communication with C. Kirschbaum^66^.

3 $$\begin{gathered} {\text{Microstructure}} = {\upbeta }_{0} { } + {\upbeta }_{1} \left( {{\text{Waist}}} \right) + {\upbeta }_{2} \left( {{\text{Age}}} \right) + {\upbeta }_{3} \left( {{\text{Cortisol}}} \right) \hfill \\ \quad \quad \quad \quad \quad \quad \quad + {\upbeta }_{4} \left( {{\text{Age }} \times {\text{Cortisol}}} \right) + {\upbeta }_{5} \left( {{\text{Waist }} \times {\text{ Cortisol}}} \right) + {\upvarepsilon } \hfill \\ \end{gathered}$$

## Data Availability

The datasets generated during and/or analysed during the current study are available from the corresponding author on reasonable request.
